# Learning curve for two-port video-assisted thoracoscopic surgery lung segmentectomy

**DOI:** 10.1093/icvts/ivab236

**Published:** 2021-09-04

**Authors:** Natasha Toleska Dimitrovska, Feichao Bao, Ping Yuan, Shoujun Hu, Xiao Chu, Wentao Li

**Affiliations:** 1 Department of Thoracic Surgery, University Clinic for Thoracic and Vascular Surgery, Skopje, Macedonia, The Former Republic of Yugoslavia; 2 Department of Thoracic Surgery, Shanghai Jiao Tong University Affiliated Chest Hospital, Shanghai, China; 3 Department of Thoracic Surgery, First Affiliated Hospital of Zhengzhou University, Zhengzhou, Henan, China; 4 Department of Thoracic Surgery, Fuyang People’s Hospital, Fuyang, Anhui, China; 5 Department of Thoracic Surgery, The Fifth People’s Hospital of Shanghai, Fudan University, Shanghai, China

**Keywords:** Learning curve, Segmentectomy, Video-assisted thoracoscopic surgery

## Abstract

**OBJECTIVES:**

When lung cancer evolves from a large, centrally located mass to small, peripherally located pulmonary nodules, such as ground glass nodules, segmentectomy offers a reasonable method by which to save lung parenchyma without eliciting compromising oncological effects. To master these techniques, it is important to analyse the learning curve of surgeons. Therefore, the aim of the present study was to analyse the learning curve for two-port video-assisted thoracoscopic surgery (VATS) segmentectomy in our institution.

**METHODS:**

We retrospectively collected data from 86 consecutive patients who underwent two-port VATS segmentectomy between June 2019 and November 2019. The operative time (OT) and estimated blood loss and other complications were analysed. The learning curve was evaluated using the OT and the cumulative sum (CUSUM) value of OTs across all cases.

**RESULTS:**

We generated a graph of the CUSUM of OTs and found that the learning curve could be differentiated into 3 phases: phase 1, the initial learning phase (1st to 27th operation); phase 2, the increased competence phase (28th to 54th operation); and phase 3, the experienced phase (55th to 86th operation). The CUSUM value inflected at patient number 47. There were significant reductions in the OT and bleeding in phase 3 relative to phases 1 and 2. There were also significant differences in OT and estimated blood loss between the simple and complex segmentectomy procedures.

**CONCLUSIONS:**

In conclusion, the 3 phases identified using CUSUM analysis of the OT represented characteristic stages of the learning curve for two-port VATS segmentectomy. The data indicate that, in our institution, the inflection point for the learning curve was achieved after operating on 47 cases.

**Figure:**
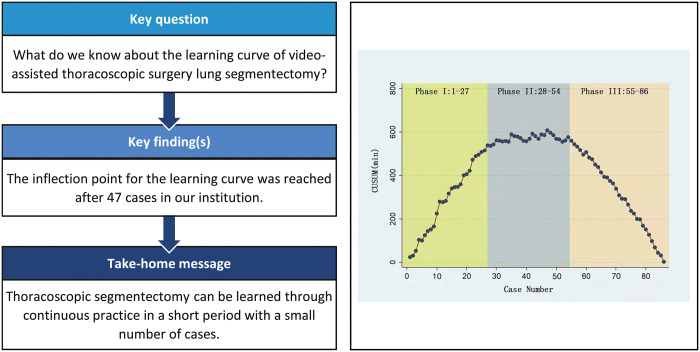

## INTRODUCTION

Lung cancer is the leading cause of cancer-related deaths worldwide. Lobectomy is the standard surgical solution for patients with early-stage lung cancer, in accordance with the results of randomized clinical trials conducted in 1994 [1, 2]. However, in cases where lung cancer evolves from a large, centrally located mass to small, peripherally located pulmonary nodules, such as ground glass nodules, segmentectomy offers a reasonable option by which to save lung parenchyma without eliciting compromising oncological effects [3–5]. The complicated anatomy of pulmonary segments makes the technique of segmentectomy difficult to study [6]. Previous publications have only vaguely described the learning curve for video-assisted thoracoscopic surgery (VATS) segmentectomy [7, 8], and the learning curve for two-port VATS segmentectomy has been limitedly studied. To master the technique for segmentectomy, it is important to analyse the learning curves of surgeons. Therefore, the aim of the present study was to analyse the learning curve for two-port VATS segmentectomy in our institution.

## METHODS

### Ethical statement

Because only de-identified data were used for the study, informed consent was waived by IRB.

We retrospectively collected data from 86 consecutive patients who underwent two-port VATS segmentectomy between June 2019 and November 2019. Two-port VATS segmentectomy was indicated for surgical resection of a target lung segment in cases involving both malignant and benign lesions. All of the operations were performed by the same junior surgeon at Shanghai Jiao Tong University Affiliated Chest Hospital. The first two-port VATS segmentectomy was performed in June 2019.

Segmentectomy procedures were subdivided based on the surgical procedure and condition of the intersegmental plane, i.e. its number and shape. Procedures such as superior, left upper division and lingual segmentectomy were considered to be simple segmentectomy techniques, while other procedures that involved intricate intersegmental planes were considered to be complex segmentectomy techniques [9].

Clinical features such as age, sex, forced expiratory volume in the first second, lesion location and size, histology, operative time (OT), estimated blood loss, conversion to multiport VATS, morbidity and mortality were recorded. OT indicates the duration of the segmentectomy procedure and does not include the durations of lymph node dissection and skin suture.

### Surgical technique of two-port video-assisted thoracoscopic surgery segmentectomy

**Figure 1:**
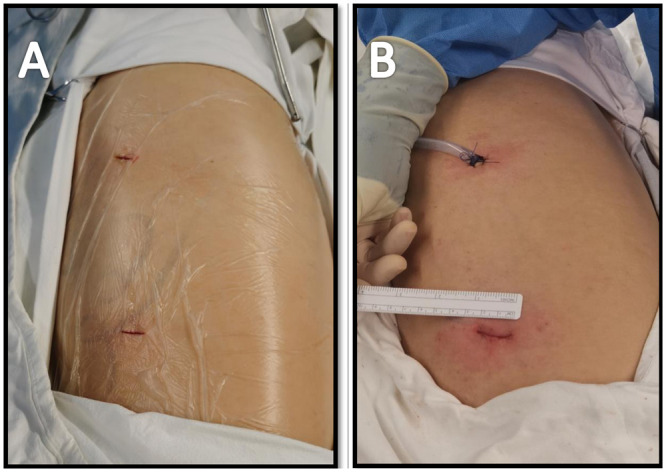
(**A**) Positions of the surgical ports for two-port video-assisted thoracoscopic surgery segmentectomy (before surgery). (**B**) Positions of the surgical ports for two-port VATS segmentectomy (after surgery).

**Figure 2:**
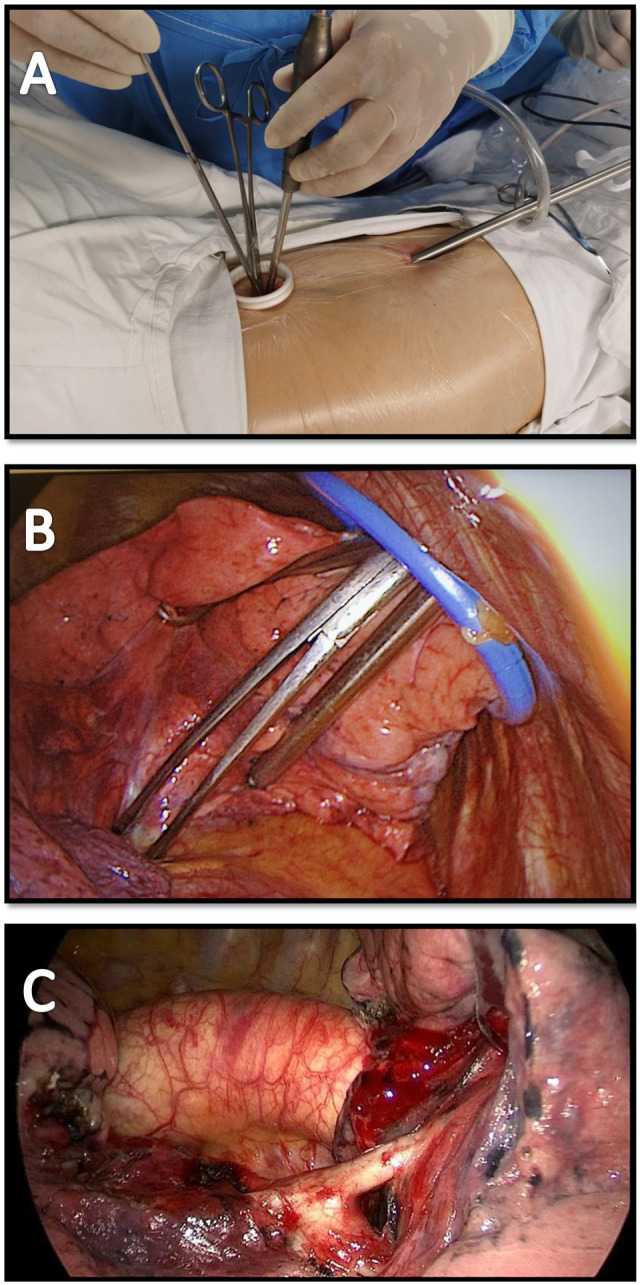
(**A**) Two-port video-assisted thoracoscopic surgery (VATS) segmentectomy (extrathoracic view). (**B**) Two-port VATS segmentectomy (intrathoracic view). (**C**) The dorsal artery of the left lower lobe was dissected in two-port VATS left lower lobe dorsal segmentectomy.

All patients received general anaesthesia through intravenous induction and were intubated with a double-lumen endotracheal tube for single lung ventilation. All patients were extubated at the end of the operation. Routine pain was managed with intercostal blocks to 2 intercostal spaces above and 2 intercostal spaces below the ports and postoperative patient-controlled analgesia. During the procedure, the patient was in the lateral decubitus position. Both the assistant and surgeon stood on the abdominal side of the patient, with the assistant standing on a footstool to operate the camera. VATS lung segmentectomy was performed using a two-port approach, with a ∼1-cm port made for the camera and a second access incision ∼2-cm long made without rib-spreading [9]. The positions of the 2 ports depended on the type of segmentectomy to be performed. In general, an 8-cm distance was maintained between the 2 ports, and a 10- to 15-cm distance was maintained from the target, which was the hilum of the lobe containing the segment to be resected (Fig. 1). We used 2 incisions with similar size to complete surgical resection (Fig. 1). VATS procedures for indeterminate nodules were performed after localization of the nodules either through the operative view or using 3-dimensional images of chest tomography. The bronchus, vein and artery were divided anatomically and transected separately using endostaplers (Covidien, Mansfield, MA, USA or Ethicon Endo-Surgery Inc., Cincinnati, OH, USA) or ligated before dissection. The intersegmental plane was then transected using an endostapler (Fig. 2 and Video 1). The specimen was removed using a surgical glove. A 28-Fr chest tube was inserted at the end of the operation.

### Statistical analysis

To analyse the mean differences between the curve phases or between operation groups, Student’s *t*-test, the Wilcoxon rank-sum test, a one-way analysis of variance or the *χ*^2^ test were used. A probability value of <0.05 was considered statistically significant. All analyses were performed using IBM Statistical Package for Social Sciences (SPSS) v20.0 (IBM Co., Armonk, NY, USA). The cumulative sum (CUSUM) method was used to quantitatively assess the learning curve [10]. CUSUM is the running total of differences between individual data points and the mean of all data points. The CUSUM for the variable of interest in the first patient was the difference between the value for the first case and the mean for all patients. The CUSUM for the second case was the sum of the CUSUM of the previous patient and the difference obtained for the second patient. This process was repeated until the CUSUM for the last case was zero. We assessed the best fit for the plot and detected the change in the slope of the CUSUM learning curve. The number of required cases was calculated from the inflection point of the curve that represented the best fit for the plot.

## RESULTS

Between June 2019 and November 2019, 86 consecutive two-port VATS segmentectomies were performed by a single surgeon. Due to intraoperative bleeding during complex segmentectomy, 4 cases were converted to multiport VATS. Postoperative complications occurred in 10 patients (11.6%), of whom 8 had a prolonged air leak lasting >7 days, 1 had pneumonia and 1 had empyema. There were no life-threatening complications in any of the cases. Neither hospital mortality nor mortality was observed within 30 days, although 1 patient (1.6%) required a 30-day readmission.

**Figure 3:**
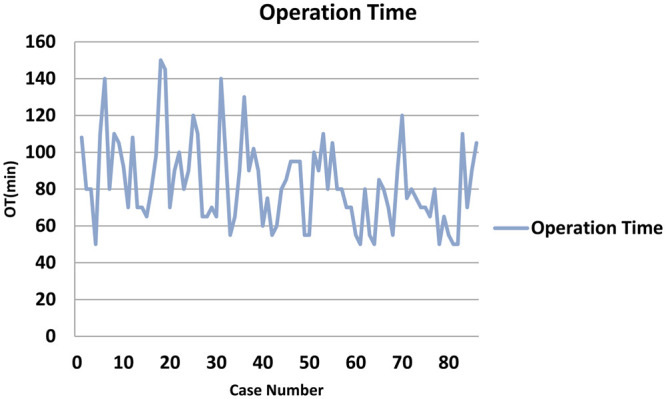
Raw OTs plotted in chronological case order. OT: operative time.

**Figure 4:**
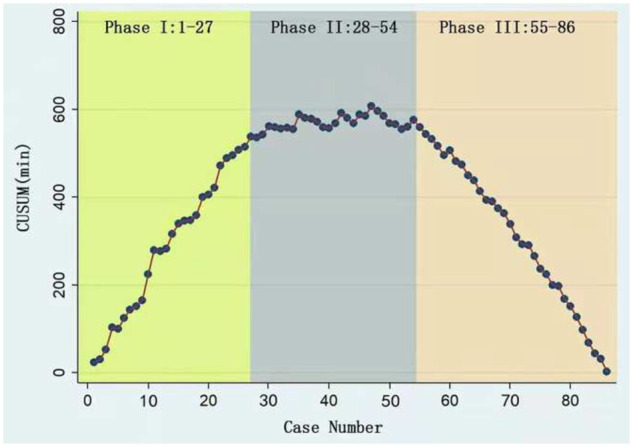
Cumulative sum plot for two-port video-assisted thoracoscopic surgery segmentectomy. Patients 1–27 constitute the ascending slope of the curve [initial learning phase (phase 1)], patients 28–54 constitute the plateau part of the curve [increased competence phase (phase 2)] and patients 55–86 constitute the descending slope [experienced phase (phase 3)].

The raw OTs of the segmentectomies were plotted in chronological case order (Fig. 3). The OT gradually decreased, but the trend was unclear. The CUSUM_OT_ learning curve was best modelled as a second-order polynomial (parabola), with the CUSUM_OT_ equation represented in min (Fig. 4). Across the 86 consecutive patients, patient number 47 represented the CUSUM value inflection point. Patients 1–27 constituted the ascending slope of the curve [initial learning phase (phase 1)], patients 28–54 constituted the plateau of the curve [increased competence phase (phase 2)] and patients 55–86 constituted the descending slope [experienced phase (phase 3)].

Comparisons of various parameters across the 3 phases identified by the CUSUM_OT_ analysis are presented in Table 1. Age, sex, comorbidity, surgical level, location and pathological diagnosis did not differ significantly across the 3 phases. There were significant reductions in the OT and blood loss in phase 3 when compared with phases 1 and 2. No significant reductions in the conversion rate were observed across the 3 phases.

**Table 1:** Interphase comparisons of patient characteristics and operative parameters in all cases

	Phase 1 (*n* = 27)	Phase 2 (*n* = 27)	Phase 3 (*n* = 32)	*P*-value
Sex, *n*				0.45
Female	15	19	18	
Male	12	8	14	
Age (years), mean ± SD	59.8 ± 11.5	57.8 ± 10.7	58.9 ± 10.0	0.80
Comorbidity, *n*				0.56
Hypertension	8	7	9	
Diabetes	6	3	4	
Others	0	2	2	
FEV1% Pred (%), mean ± SD	87.2 ± 23.3	85.7 ± 27.0	83.8 ± 14.8	0.51
Surgical level, *n*				0.71
Simple	11	9	14	
Complex	16	18	18	
OT (min), mean ± SD	105.5 ± 20.0	84.6 ± 14.8	62.8 ± 11.1	<0.001
EBL (ml), mean ± SD	206.7 ± 63.3	173.3 ± 69.9	125.0 ± 67.2	<0.001
Conversion, *n*	2	0	2	0.38
Morbidity, *n*	3	4	3	0.84
Mortality, *n*	0	0	0	NA
Pathology, *n*				0.90
Malignant	24	25	29	
Benign	3	2	3	
Oncological status, *n*				0.93
AIS	10	7	13	
MIA	7	9	5	
IAC	6	7	9	
SCC	1	2	2	
AAH	1	1	2	
Tuberculoma	2	1	1	
Tumour size, mean ± SD	1.89 ± 1.13	1.81 ± 1.02	1.73 ± 1.15	0.87

AAH: atypical adenomatous hyperplasia; AIS: adenocarcinoma *in situ*; EBL: estimated blood loss; FEV: forced expiratory volume; IAC: invasive adenocarcinoma; MIA: microinvasive adenocarcinoma; NA: not applicable; OT: operative time; SCC: squamous cell carcinoma.

When the characteristics of patients who underwent simple and complex segmentectomy were compared, we found significant differences in OT and estimated blood loss, but not in conversion, morbidity and mortality (Table 2). The mean OT of simple segmentectomy was 73.7 min, which was much shorter than that of complex segmentectomy (89.1 min; *P* = 0.003).

**Table 2:** Comparison of patient characteristics and operative parameters between cases of simple and complex segmentectomy

	Simple	Complex	*P*-value
Sex, *n*			
Male	18	16	
Female	16	36	0.067
Age (years), mean ± SD	56.8 ± 8.6	60.2 ± 11.6	0.15
Comorbidity, *n*			0.85
Hypertension	9	15	
Diabetes	3	9	
Others	1	3	
FEV1% Pred (%), mean ± SD	95.0 ± 17.0	85.9 ± 25.6	0.24
OT (min), mean ± SD	73.7 ± 16.7	89.1 ± 25.3	0.003
EBL (ml), mean ± SD	139.7 ± 53.3	182.9 ± 81.5	0.007
Conversion, *n*	0	4	0.26
Morbidity, *n*	3	7	0.76
Mortality, *n*	0	0	NA
Oncological status, *n*			0.73
AIS	13	17	
MIA	8	13	
IAC	6	16	
SCC	3	2	
AAH	2	2	
Tuberculoma	2	2	
Tumour size, mean ± SD	1.70 ± 1.33	1.97 ± 0.91	0.48

AAH: atypical adenomatous hyperplasia; AIS: adenocarcinoma *in situ*; EBL: estimated blood loss; FEV: forced expiratory volume; IAC: invasive adenocarcinoma; MIA: microinvasive adenocarcinoma; NA: not applicable; OT: operative time; SCC: squamous cell carcinoma.

## DISCUSSION

The widespread use of computed tomography for lung cancer screening has increased the frequency of identification of early-stage lung cancer [11]. Consequently, segmentectomy has emerged as a viable option to save the pulmonary parenchyma. However, segmentectomy is not yet widely accepted due not only to its controversial oncological effects and associated morbidity but also to the technical difficulty of the procedure, which is exacerbated by anatomical variations and the large size of the dissection plane [12, 13].

We analysed the learning curve of this two-port VATS for segmentectomy. The learning curves of VATS and robotic surgery for segmentectomy have only been vaguely described in previous publication [7, 8, 14]. Our study suggests that thoracic surgeons with skilled VATS techniques require relatively short periods to learn the two-port VATS segmentectomy procedure. This short learning curve can be explained by several reasons. First, the surgeon may be skilled in VATS surgery based on previous experience of both wedge and lobectomy, familiarity with the thoracoscopic appearance of pulmonary anatomy would be particularly helpful in this regard. Second, the 86 segmentectomies were performed within a relatively short period compared with the periods of other studies [7], and it is known that repeated practice within a short period enables surgeons to better master complicated procedures.

Different kinds of thoracoscopic techniques have been applied for segmentectomy [15–17]. Theoretically, the single-port VATS was the least invasive technique, but it does have some disadvantages. First, the chest tube is placed between the fourth of fifth intercostal space, which may not conducive to the drainage of pleural effusion. Second, camera and multiple instruments were introduced via the single incision, and repeated compression to intercostal nerve may increase postoperative pain. Third, the single-port VATS is technical demanding, which is not beneficial for its widespread application. After attempting different thoracoscopic techniques, we adopted the two-port VATS surgery, which is evolved from the conventional two-port thoracoscopic surgery, and we use 2 incisions, which is the quite smaller when compared with published literatures [17, 18]. The utility incision was ∼2 cm in length, which seems difficult for specimen retrieval and multiple instrument introduction. By using surgical wound protector, 3 or more articulated instruments could be introduced simultaneously in the same incision, and specimen of segment was much smaller when compared with lobe, it would not be a problem to retrieve the specimen from the small incision.

We did not use skin-to-skin time to analyse the learning curve, as the types of lymphadenectomies performed varied across patients with different kinds of disease. Some patients with invasive adenocarcinoma required systematic lymph node dissection or sampling, whereas others with less-invasive cancer like minimally invasive adenocarcinoma required selective lymph node dissection or sampling. Therefore, the duration of segmentectomy was a more precise marker for analysis of the learning curve. Complex segmentectomy is an intricate procedure that requires the isolation and division of suitable segmental bronchi, arteries and veins more peripherally and the construction of several intersegmental planes [9]. It would therefore be relatively easy to understand increased OT and estimated blood loss in complex segmentectomy when compared with simple segmentectomy. Theoretically, the perceived complexity of complex relative to simple segmentectomy ought to have increased morbidity factors such as persistent air leaks; however, we did not observe any such increases, it may due to small case volume included for this study. Four cases need conversion, as the dissection of anatomical structures in segmentectomy is more distal than for lobectomy. The vessel injured during segmentectomy would be much smaller than that in lobectomy, most of the bleeding were not catastrophic, which could be controlled with an additional port rather than thoracotomy.

Previously, some reports have been focused on study for the learning curve of VATS segmentectomy [7, 8]. Cheng studied the feasibility and learning curve of single-port VATS segmentectomy, and the study with 40 cases included suggested that single-port VATS is a safe and feasible technique with acceptable morbidity and mortality [8]. In another study with more cases included, the learning curve was much longer, which may because the time period of the study is over 10 years. In our study, the learning curve of small double port VATS segmentectomy is shorter, it may due to repeated training in a short time period and simple segmentectomy and complex segmentectomy were also compared in our study.

### Limitations

The primary limitation of this study is that it is a single-centre retrospective study with limited case numbers. Apart from operation time, morbidity, mortality conversion rate and blood loss can also be good markers for the difficulty of operation, but due to the small sample size, and no significant difference was found between different phases of learning curve. Several factors would influence the result of the study, which are listed below: the surgeon’s previous experience in VATS, the selection of patients, the difficulty of the procedure caused by anatomic variations, calcified hilar lymph nodes, incomplete fissure and the status of surgical team, especially the camera assistant. Although we divided segmentectomy into simple and complex segmentectomy, because of limited included patients, we still cannot analyse the learning curve separately according to the factors mentioned above. Other parameters including chest tube drainage duration, length of hospital stay and long-term oncology outcomes should be analysed in future prospective multicentre studies.

## CONCLUSION

In conclusion, the 3 phases identified using CUSUM analysis of the operation time represented characteristic stages of the learning curve for two-port VATS segmentectomy. The data indicate that, in our institution, the inflection point for the learning curve was achieved after operating on 47 cases.

## Funding

The study was partly funded by the National Natural Science Foundation of China [81900099], Wu Jieping Medical Foundation [320.320.2730.1869] and Talent Development Plan funded by Shanghai Fifth People’s Hospital, Fudan University [2020WYRCJY06].

**Conflict of interest:** none declared.

## ABBREVIATIONS

CUSUM: Cumulative sum
OT: Operative time
VATS: Video-assisted thoracoscopic surgery
